# Diagnostic efficacy of smear plus liquid-based cytology for EUS-FNA of solid pancreatic lesions: A propensity-matched study

**DOI:** 10.1097/MD.0000000000015575

**Published:** 2019-05-13

**Authors:** Masahiro Itonaga, Shin-Ichi Murata, Keiichi Hatamaru, Takashi Tamura, Junya Nuta, Yuki Kawaji, Takao Maekita, Mikitaka Iguchi, Jun Kato, Fumiyoshi Kojima, Hiroki Yamaue, Manabu Kawai, Ken-Ichi Okada, Seiko Hirono, Toshio Shimokawa, Kensuke Tanioka, Masayuki Kitano

**Affiliations:** a Second Department of Internal Medicine; b Department of Human Pathology; c Second Department of Surgery; d Clinical Study Support Center, Wakayama Medical University, Wakayama, Japan.

**Keywords:** EUS-guided fine needle aspiration, liquid-based cytology, propensity score-matching, smear cytology, solid pancreatic lesion

## Abstract

Liquid-based cytology (LBC) is a thin-layer slide preparation procedure that was developed to overcome the cell crowding and contamination associated with smear cytology (SC). The present study compared diagnostic efficacy between SC alone and SC combined with LBC (SLBC) using endoscopic ultrasound-guided fine needle aspiration (EUS-FNA) samples of pancreatic lesions.

We retrospectively analyzed data derived from 311 consecutive patients. Specimens obtained via EUS-FNA from 179 patients between December 2011 and May 2016 were analyzed by SC, and those obtained from 132 patients between June 2016 and October 2017 were analyzed by SLBC. The 2 groups were compared in terms of adequate sample rate, diagnostic accuracy, sensitivity, and specificity using propensity score matching.

SC and SLBC were compared using propensity score-matching in 204 patients (n = 102 per group). The adequate sample rate did not differ significantly between SLBC (100%) and SC (99.0%, *P* = 1). Diagnostic sensitivity, negative predictive value and accuracy were better for SLBC than for SC in terms of cytological (93.2% vs 67.4%, 68.4% vs 23.1%, and 94.1% vs 69.6%, *P* < .01 each, respectively) and cytohistological (95.5% vs 81.5%, 76.5% vs 34.6%, and 96.1% vs 82.4%, *P* < .01, *P* = .02, and *P* < .01, respectively) analyses.

SLBC improves the diagnostic efficacy of EUS-FNA for pancreatic lesions compared to LBC.

## 1 Introduction

Endoscopic ultrasound-guided fine needle aspiration (EUS-FNA) is widely applied to the histological diagnosis of abdominal tumors, particularly of pancreatic lesions.^[1,2]^ The diagnostic performance of this technique varies depending on several factors, including tumor size, tumor location, and tumor characteristics.^[3]^ Several device characteristics and methods, such as needle size and form, suction, slow-pull and fanning techniques as well as rapid on-site cytological evaluation (ROSE) are important for the accurate diagnosis of small samples.^[4–8]^ Specimens collected by EUS-FNA have traditionally been analyzed using smear cytology (SC), which has become the standard method of cytological diagnosis. However, SC shows some disadvantages, such as cell crowding and blood contamination. Liquid-based cytology (LBC) is a thin-layer slide preparation procedure that was developed to overcome the cell crowding and contamination issues associated with SC.^[9]^ The diagnostic value of cervical cytology uterine cervical cancer using LBC is now established worldwide^[10]^ and breast cancer,^[11]^ thyroid cancer,^[12]^ and lymphoma^[13]^ have been assessed using LBC. However, comparisons of diagnostic accuracy between SC and LBC for various diseases, including pancreatic lesions,^[14–21]^ have yielded controversial findings. Moreover, no reports have compared EUS-FNA results between SC alone and SC combined with LBC (SLBC) for pancreatic lesions. The present study, therefore, compared diagnostic efficacy between SC alone and SLBC using EUS-FNA samples of pancreatic lesions and propensity score matching.

## 2 Patients and methods

### 2.1 Patients

This retrospective study evaluated data from 311 consecutive patients who provided written informed consent to undergo initial EUS-FNA for suspected solid pancreatic lesions at Wakayama Medical University Hospital between December 2011 and October 2017. We used SC and SLBC to analyze EUS-FNA specimens from 179 patients investigated between December 2011 and May 2016 and from 132 patients investigated between June 2016 and October 2017, respectively. This retrospective study was approved by the ethics committee at Wakayama Medical University Hospital and proceeded in accordance with the Declaration of Helsinki.

### 2.2 EUS-FNA procedure

Patients underwent EUS-FNA using a GF-UCT260 linear echoendoscope (Olympus Medical, Japan) connected to a Prosound α-10 ultrasound scanning system (Hitachi Aloka Medical, Japan), an EU-ME-2 universal ultrasound processor (Olympus Medical) and Expect (Boston Scientific Corp, Natick, MA) or EZ Shot2 (Olympus Medical) 19-, 22-, and 25-gauge FNA needles. Lesions located in the head of the pancreas were sampled using a transduodenal approach, while those in the pancreatic body or tail were obtained via a transgastric approach. Pancreatic lesions were detected after EUS evaluation and punctured using 1 of the 3 types of needles. Thereafter, 20 to-and-fro movements within the lesion proceeded with 20 mL of negative pressure until a sufficient amount of material was obtained for ROSE by endosonographers who had been performing ROSE at our institution for 3 years, since learning this technique from a cytopathologist.

### 2.3 Smear cytology

**Figure 1:**
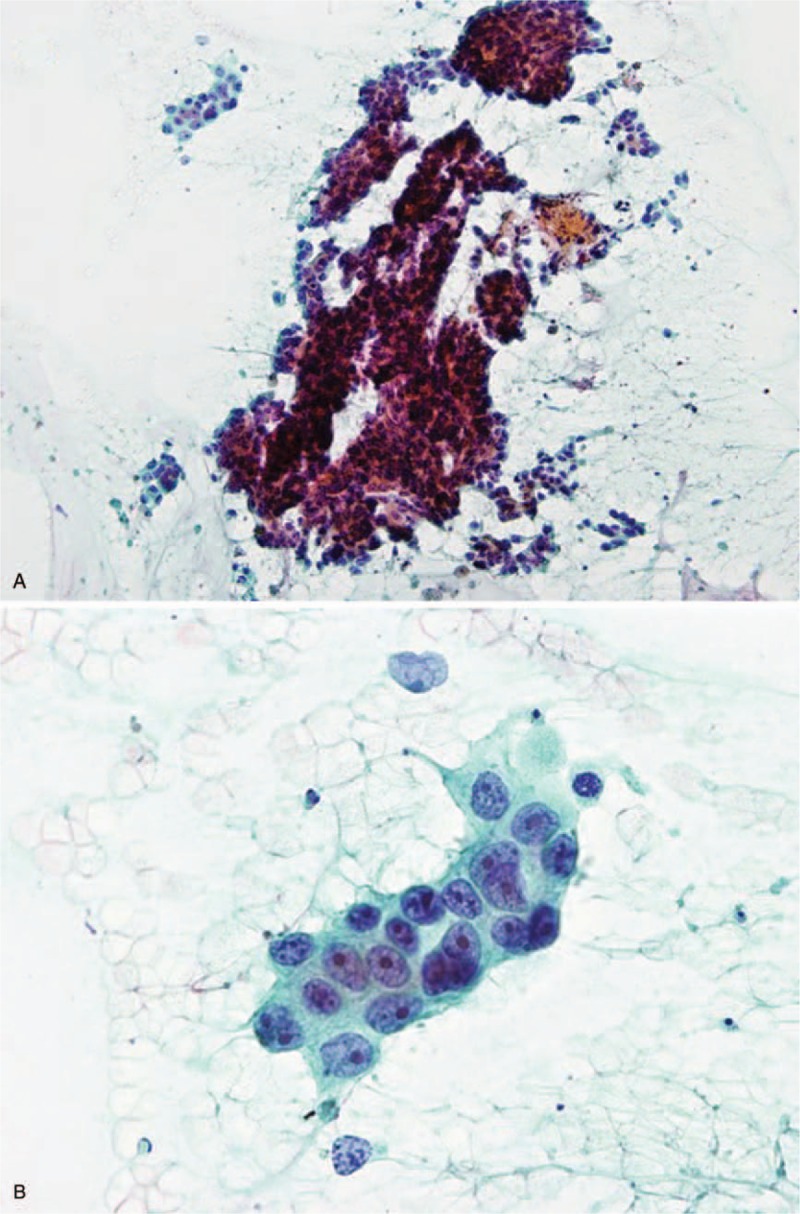
Conventional smear images demonstrating pancreatic ductal adenocarcinoma (A: ×100, B: ×400).

Two cytological smears were prepared from each specimen, with the remainder placed in formalin for histological analysis. One of the 2 cytological smears was air-dried for Diff-quick and the other was wet-fixed with an ethanol-based fixative for Papanicolaou staining in the Department of Pathology (Fig. 1).

### 2.4 Liquid-based cytology (Fig. 2)

**Figure 2:**
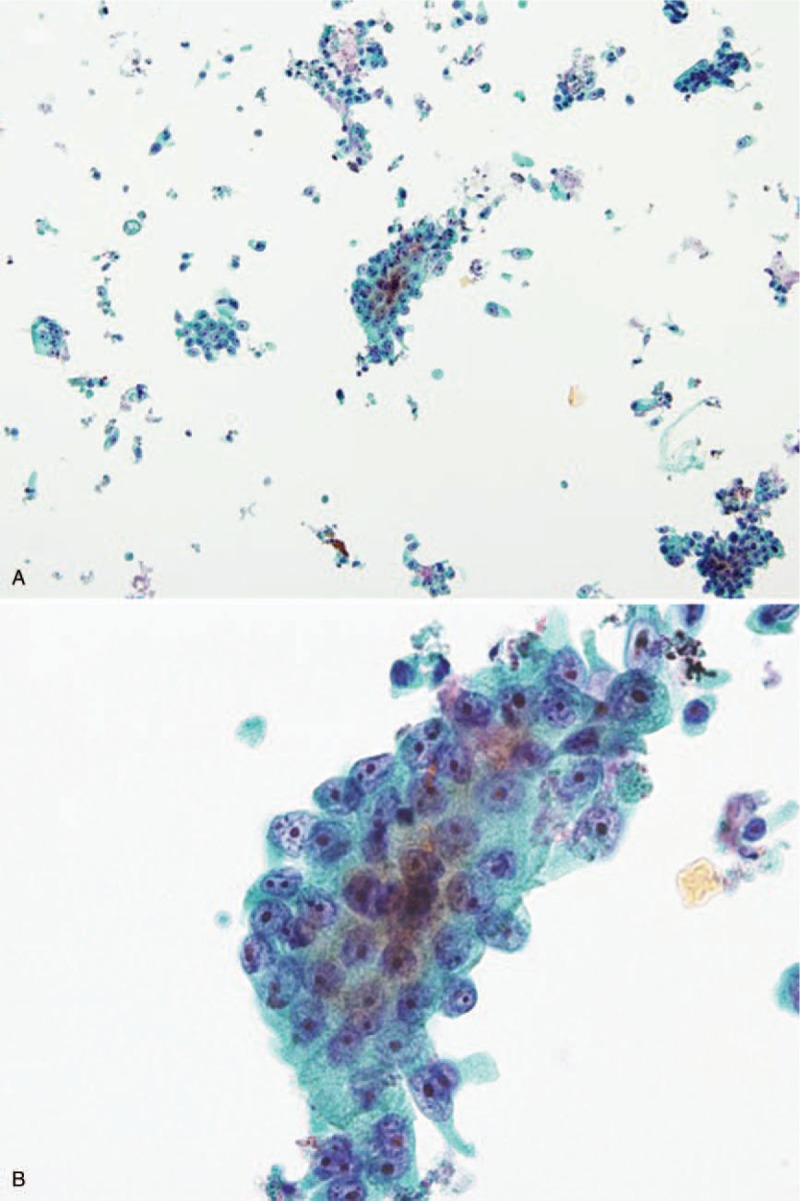
Liquid-based cytology images demonstrating pancreatic ductal adenocarcinoma (A: ×100, B: ×40).

After extracting visible tissue for SC and histological analysis, residual specimens in the FNA needle were collected into fixation medium for LBC analysis (ThinPrep System) in the Department of Pathology. Cells were isolated from the fluid by vacuum filtration and were transferred to the slide using air pressure for adherence. Slides for LBC were prepared and fixed in 95% ethanol for 24 to 48 hours. These slides were stained using the Papanicolaou procedure and examined under light microscopy (Fig. 2).^[18]^

### 2.5 Histological analysis

Specimens acquired by EUS-FNA were dropped onto glass slides. Visible tissue was selected, fixed in 10% formalin, embedded in paraffin and thinly sliced for additional immunostaining as required.

### 2.6 Definitions

An experienced cytotechnologist and a pathologist assessed and diagnosed the specimens. Cytological and histological diagnoses were classified as negative for malignancy (“negative”), atypical, suspected malignancy (“suspected”), positive for malignancy (“positive”) or inadequate. Malignancy was therefore defined as suspected or positive, and benignancy as negative or atypical. Neuroendocrine tumor (G1 or G2) and solid-pseudopapillary neoplasm were defined as benign in this study. SLBC results were based on combined SC and LBC findings, and cytohistological results were based on combined cytological and histological findings. The final diagnosis was confirmed based on the clinical course during the subsequent 12 months and cytohistological analysis after surgical resection.

### 2.7 Statistical methods

The primary aim of this study was to clarify whether SLBC was superior compared to SC. However, factors contributing to patient background variability included age, sex, location of lesion, lesion size, FNA needle size, number of needles, final diagnosis and adverse events. To reduce these differences between the SLBC group and the SC group, 1-to-1 propensity score matching was used. Propensity scores were calculated by logistic regression, and matching was conducted as nearest neighbor matching with a caliper coefficient of 0.2.

For these matched patients, sensitivity, specificity, positive predictive value, negative predictive value, and accuracy were compared between the SC group and the SLBC group, using the chi-squared test with an alpha value of 0.05. In addition, 95% confidence intervals for the ratio were calculated. In the same manner, these tests were conducted to compare SC with histology and SLBC with histology.

To describe the background of patients, the significance of differences in continuous data was assessed using paired or nonpaired Student *t* tests as a reference. On the other hand, chi-squared tests were used for analyzing qualitative data. Data were statistically analyzed using JMP Pro version 12 (SAS Institute Inc, Cary, NC)

## 3 Results

Table 1 shows patient characteristics. A total of 179 patients were enrolled in the SC group, and 132 patients into the SLBC group. Groups differed significantly in terms of mean patient age, FNA needle size and mean number of needle passes (SC group vs SLBC group: 65.5 years vs 70.7 years, *P* < .01, 19 G 5.0%, 22 G 89.4%, 25 G 5.6% vs 19 G 0.7%, 22 G 97.0%, 25 G 2.2%, *P* = .03, and 3.4 vs 2.8, *P* < .01 respectively). The percentage of patients who underwent surgery was 22.1% (69 patients). We followed-up the remaining 242 patients who did not undergo surgery for at least 12 months or until the death of the patient. The final diagnosis in 221 patients was malignancy based on disease progression during follow-up or apparent distant metastases on computed tomography (CT) (and/or magnetic resonance imaging [MRI] and/or EUS). The final diagnosis in 21 patients was benignancy based on unchanged shape and size on CT (and/or MRI and/or EUS) during follow-up. We analyzed data from 204 patients (n = 102 per group) for propensity score-matching (Table 2). Table 3 shows the cytological outcomes for malignant and benign lesions. Lesions in the SC group were diagnosed as negative in 8, atypical in 22, suspected in 16 and positive in 46 (total, n = 92). Ten lesions were diagnosed as benign, including 4 negative, 5 atypical, and 1 inadequate sample. Malignant lesions were diagnosed in 90 patients in the SLBC group comprised 6 atypical, 18 suspected, and 66 positive samples. Twelve lesions were diagnosed as benign, including 5 negative and 7 atypical samples. Adequate sample rate did not differ between the SC group (99.0%) and SLBC group (100%, *P* = 1).

**Table 1:**
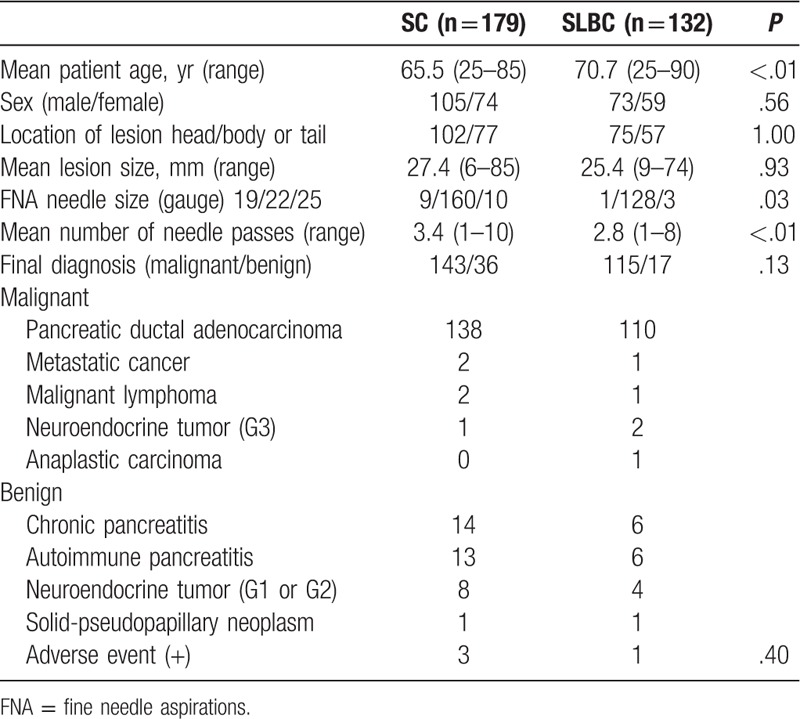
Patient characteristics.

**Table 2:**
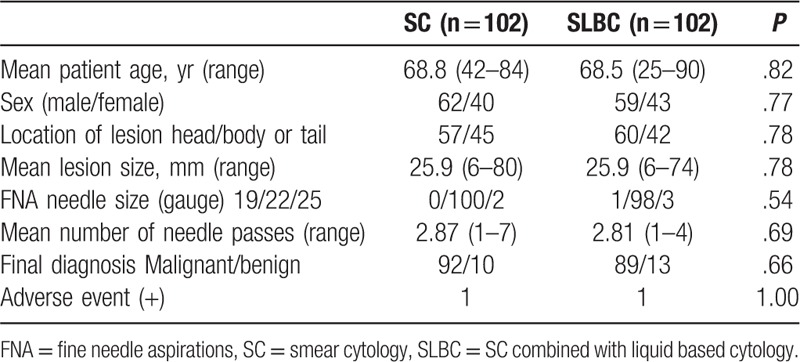
Comparison of demographics between smear cytology (SC) and SC combined with liquid-based cytology (SLBC) using propensity score matching.

**Table 3:**
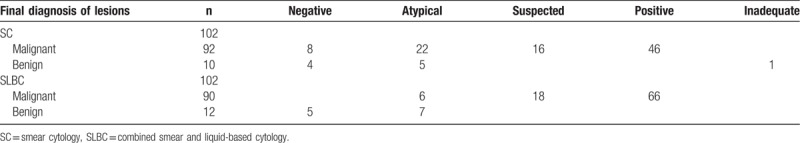
Cytological outcomes of malignant and benign lesions in propensity-matched groups.

Table 4 compares diagnostic yields between SC and SLBC. Sensitivity, negative predictive values, and accuracy of cytological diagnosis of malignancy were significantly higher in the SLBC group than in the SC group (93.2% vs 67.4%, 68.4% vs 23.1%, and 94.1% vs 69.6%, *P* < .01, respectively), whereas differences in specificity and positive predictive value were not significant. Sensitivity, negative predictive value, and accuracy of cytohistological diagnosis of malignancy were significantly higher in the SLBC group than in the SC group (95.5% vs 81.5%, 76.5% vs 34.6%, and 96.1% vs 82.4%, *P* < .01, *P* = .02, and *P* < .01, respectively).

**Table 4:**
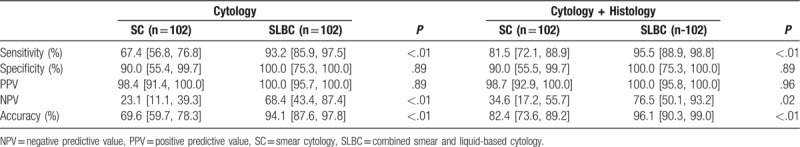
Comparison of diagnostic yields between smear cytology and combined smear and liquid-based cytology.

## 4 Discussion

The present study compared the diagnostic performance of SC and SLBC for pancreatic samples obtained by EUS-FNA. Direct comparisons between the SC and SLBC groups using propensity score-matching demonstrated that diagnostic sensitivity, negative predictive values, and accuracy were higher in the SLBC group than in the SC group. This report is the first to compare EUS-FNA the results of SC alone and SLBC for pancreatic lesion and to demonstrate that SLBC contributes additional diagnostic efficacy to EUS-FNA for pancreatic lesions.

Today, we can use SC, LBC, and cell block (CB) as cytological methods in EUS-FNA. Each of these 3 methods has specific advantages and disadvantages.

SC is an established method for diagnosing EUS-FNA samples of pancreatic lesions. Despite adequate performance, an important limitation of SC is that samples often become dehydrated during ethanol fixation. In this study, 1 sample was inadequately diagnosed in our study due to dehydration.

The CB method allows cytological and/or histological evaluation with the hematoxylin and eosin staining that is so familiar to pathologists, and with immunostaining of serial sections if necessary. However, the CB method decreases the number of cells during the complicated preparation procedures and therefore a much smaller number of cells can be observed for examination compared to SC.^[22]^

LBC has overcome the drawbacks of cell crowding and blood contamination in SC using a single layer of cells.^[9]^ Samples from patients with cervical cancer,^[10]^ breast cancer,^[11]^ thyroid cancer,^[12]^ and lymphoma^[13]^ have been cytologically assessed using LBC, which offers several advantages over SC. One advantage is that this method significantly enhances specimen adequacy by reducing the number of inadequate diagnoses due to ambiguities caused by inflammation, blood contamination, and poor fixation. Another advantage is that samples prepared for LBC can be saved for later cytological reanalysis, immunostaining, and genetic testing.^[23,24]^ However, the disadvantages are the more labor-intensive methods, increased cost of preparing cytological specimens and steep learning curve to familiarize cytopathologists with the slides.^[25]^

The results of previous comparisons of diagnostic performance between SC and LBC for various diseases are controversial. Siebers et al^[14]^ reported that the performance of LBC in terms of relative sensitivity and PPV for detecting cervical cancer precursors was not any better than that of conventional SC. In contrast, Son et al^[15]^ found that LBC could reveal more cellularity with a cleaner background and better cytomorphological features and deliver markedly higher diagnostic sensitivity than SC. Few studies have compared diagnostic performance between SC and LBC with respect to EUS-FNA samples of pancreatic lesions. LeBlanc et al^[18]^ and Hashimoto et al^[19]^ found relatively higher diagnostic performance for LBC compared with SC for EUS-FNA samples of pancreatic lesions. Qin et al^[20]^ reported relatively higher diagnostic performance for LBC compared with SC, but the difference was not significant. On the other hand, Yeon et al^[21]^ reported LBC showed lower diagnostic accuracy for pancreatic EUS-FNA compared with SC.

The present comparison of diagnostic efficacy between SLBC and SC showed that SLBC increases the diagnostic efficacy of EUS-FNA for pancreatic lesions. Kim et al^[26]^ reported that the ability of SLBC to diagnose thyroid fine-needle aspiration samples was significantly better than that of SC. The advantages of SLBC are as follows. First, the 2 types of slides prepared for SLBC allow a reduction in the number of inadequate samples and facilitates cytological diagnosis. Moreover, in LBC analysis, diagnosis can be achieved from residual specimens after visible tissues are extracted and fixed in formalin solution for histological analysis. Second, in this study, the mean number of needle passes was lower in the SLBC group than in the LBC group. The number of punctures may be able to be reduced using SLBC.

Our study has the following limitations. First, the study used a retrospective design, implemented at a single center, and with a small sample size. The 2 groups differed significantly in terms of mean patient age, FNA needle size, and mean number of needle passes as patient characteristics. We adopted propensity score-matching to reduce potential sources of bias. However, complete elimination of bias seems impossible. A prospective multicenter study is necessary to confirm our conclusion. Second, ROSE was performed only by endosonographers to ensure tissue adequacy. ROSE was reported to increase diagnostic accuracy from 69.2% in the period without ROSE by endosonographers to 91.8% in period with ROSE by endosonographers.^[27]^ However, our results for ROSE were not as accurate as described in that previous study. Twenty-two malignant lesions (23.9%) were diagnosed as “atypical” in adequate samples in the SC group, while 6 malignant lesions (6.7%) were diagnosed as “atypical” in adequate samples in the SLBC group. In the SC group, sensitivity was low because we defined findings of “atypical” as benign. However, in the SLBC group, the 2 types of slides prepared for SLBC allowed a reduction in the number of “false-negative” samples (“negative” and “atypical” in samples with a final diagnosis of malignant) and facilitated cytological diagnosis. Third, this study lacked a cost-benefit analysis. Adding LBC to SC increases costs compared with SC alone. Finally, only 69 patients (22.1%) underwent surgery, although final diagnoses were made according to the clinical course for 12 months in the remaining patients.

In conclusion, SLBC improves the diagnostic efficacy of EUS-FNA compared with LBC for solid pancreatic lesions.

## Author contributions

Itonaga M, Murata S, and Kitano M designed the research. Itonaga M, Tamura T, Nuta J, Maekita T, Iguchi M, Tamai H, Kato J, Kojima F, Yamaue H, Kawai M, Okada KI, and Hirono S contributed case registration. Shimokawa T and Tanioka K analyzed the data. Itonaga M drafted the manuscript. All authors have read and approved the final version to be published.

**Data curation:** Masahiro Itonaga, Shin-Ichi Murata, Keiichi Hatamaru, Takashi Tamura, Junya Nuta, Yuki Kawaji, Takao Maekita, Mikitaka Iguchi, Jun Kato, Fumiyoshi Kojima, Hiroki Yamaue, Manabu Kawai, Ken-Ichi Okada, Seiko Hirono, Kensuke Tanioka, Masayuki Kitano.

**Formal analysis:** Toshio Shimokawa, Kensuke Tanioka.

**Methodology:** Masahiro Itonaga, Toshio Shimokawa.

**Project administration:** Masahiro Itonaga.

**Software:** Toshio Shimokawa, Kensuke Tanioka.

**Validation:** Manabu Kawai.

**Visualization:** Masahiro Itonaga.

**Writing – original draft:** Masahiro Itonaga.

**Writing – review and editing:** Masayuki Kitano.
